# Improving vascular maturation using noncoding RNAs increases antitumor effect of chemotherapy

**DOI:** 10.1172/jci.insight.149896

**Published:** 2021-04-08

**Authors:** Lingegowda S. Mangala, Hongyu Wang, Dahai Jiang, Sherry Y. Wu, Anoma Somasunderam, David E. Volk, Ganesh L. R. Lokesh, Xin Li, Sunila Pradeep, Xianbin Yang, Monika Haemmerle, Cristian Rodriguez-Aguayo, Archana S Nagaraja, Rajesha Rupaimoole, Emine Bayraktar, Recep Bayraktar, Li Li, Takemi Tanaka, Wei Hu, Cristina Ivan, Kshipra M Gharpure, Michael H. McGuire, Varatharasa Thiviyanathan, Xinna Zhang, Sourindra N. Maiti, Nataliya Bulayeva, Hyun-Jin Choi, Piotr L. Dorniak, Laurence J.N. Cooper, Kevin P. Rosenblatt, Gabriel Lopez-Berestein, David G. Gorenstein, Anil K. Sood

Original citation: *JCI Insight*. 2016;1(17):e87754. https://doi.org/10.1172/jci.insight.87754

Citation for this corrigendum: *JCI Insight*. 2021;6(7):e149896. https://doi.org/10.1172/jci.insight.149896

The Editors previously posted an Expression of Concern for this article regarding images in Supplemental 4B that appeared similar (Endo40 and Endo42 samples) and images in Supplemental Figure 9 that appeared to be the same (CH and CH/Endo28-NPs for heart tissue). No changes were necessary for Supplemental Figure 4B, as the Endo40 and Endo42 samples were determined to be derived from adjacent tumor sections. An institutional review committee concluded that the error in Supplemental Figure 9 was inadvertent and recommended correction. The authors provided the correct image for the CH/Endo28-NPs sample from the original study. An updated supplemental file that includes the correct version of Supplemental Figure 9 is now posted.

The authors regret the error.

## Supplementary Material

Supplemental data

## Version 1 04/01/2021

In-Press Preview

## Version 2 04/08/2021

Electronic publication

## Version 2 04/21/2021

Electronic publication.

## Version 3 04/21/2021

Electronic publication.

