# Supplementary material for: Improving vascular maturation using noncoding RNAs increases antitumor effect of chemotherapy
Source: JCI Insight. 2021 Apr 8;6(7):e149896. doi: 10.1172/jci.insight.149896 (PMC8119196; doi:10.1172/jci.insight.149896)
Supplement: Supplemental data [file jciinsight-6-149896-s003.pdf]

Improving vascular maturation using non-coding RNAs increases anti-tumor effect of chemotherapy

Lingegowda S. Mangala, Hongyu Wang, Dahai Jiang, Sherry Y. Wu, Anoma Somasunderam, David E. Volk, Ganesh L. R. Lokesh, Xin Li, Sunila Pradeep, Xianbin Yang, Monika Haemmerle, Cristian Rodriguez-Aguayo, Archana S Nagaraja, Rajesha Rupaimoole, Emine Bayraktar, Recep Bayraktar, Li Li, Takemi Tanaka, Wei Hu, Cristina Ivan, Kshipra M Gharpure, Michael H. McGuire, Varatharasa Thiviyanathan, Xinna Zhang, Sourindra N. Maiti, Nataliya Bulayeva, Hyun-Jin Choi, Piotr L. Dorniak, Laurence J.N. Cooper, Kevin P. Rosenblatt, Gabriel Lopez-Berestein, David G. Gorenstein and Anil K. Sood.

Supplemental Materials and Methods

siRNA and nanoparticles (NPs)

Non-silencing control siRNA, Alexa 488-labeled control siRNA, annexin A2, TEM7 siRNAs, chitosan (CH; molecular weight 50-190 kDa), sodium triphosphate (TPP), and agarose were purchased from Sigma-Aldrich (St. Louis, MO). MiR-inhibitors were purchased from Ambion (Austin, TX).

Cell lines and culture

The human epithelial ovarian cancer cell lines HeyA8 and SKOV3ip1 were maintained as described previously (1). Human immortalized umbilical endothelial cells, RF24, were a kind gift from Dr. Lee M. Ellis, Department of Surgical Oncology, The University of Texas MD Anderson Cancer Center, Houston, and were grown in modified Eagle medium (MEM) with supplements (sodium pyruvate, non-essential amino acids, MEM vitamins, and glutamine). G1S1 skin endothelial cells (ECs) were a kind gift from Dontscho Kerjaschki from the Institute of Pathology at the Medical University of Vienna. These cells were grown in EBM-2 with supplements (Lonza, Walkersville, MD) (2). The derivation and characterization of mouse ovarian endothelial cells (MOEC) was described previously (3). Human microvascular endothelial cells (HMVEC) were a kind gift from Dr. Rong Shao at the University of Massachusetts, Massachusetts. These were grown on flasks coated with

attachment factors in M131 medium with microvascular growth supplement (Life Technologies, Grand Island, NY). Cell cultures were maintained at 37°C in a 5% CO_2 incubator with 95% humidity. For *in vivo* injections, cells were trypsinized and centrifuged at 1,200 rotations per minute (rpm) for 5 min at 4°C, washed twice with phosphate buffered saline (PBS), and reconstituted in serum-free Hank's balanced salt solution (Life Technologies). Only single-cell suspensions with more than 95% viability (as determined by trypan blue exclusion) were used for *in vivo* intraperitoneal injections.

Silencing of miRNAs in ECs

We silenced miRNAs in normal ECs (RF24 or G1S1) using miR-inhibitors. Briefly, cells were transfected with either control or targeted miR-inhibitors using RNAiFect transfection reagent (Qiagen) and serum-free medium (SFM). After 6 h of transfection, either MEM or EBM2 complete medium was added and incubated for 8 h. The complete medium was replaced with conditioned medium from ovarian cancer SKOV3ip1 cells to mimic the tumor microenvironment for normal ECs. After 36 h of transfection, the expression of

miRNAs and tight junction proteins was determined using quantitative real time PCR (q-RT-PCR).

Quantitative real-time PCR validation

Quantitative real-time RT-PCR was performed using 50 ng of total RNA from purified endothelial cells was isolated using the RNeasy mini kit (Qiagen) according to the manufacturer's instructions. Complementary DNA (cDNA) was synthesized from 0.5-1μg of total RNA using Verso cDNA kit (Thermo Scientific). Quantitative PCR (qPCR) analysis was performed in triplicate using the SYBR Green ER qPCR SuperMix Universal (Invitrogen) and Bio-Rad (Bio-Rad Laboratories, Hercules, CA). Relative quantification was calculated using the $2^{-\Delta\Delta CT}$ method normalizing to control for percent fold changes (4).

Western blotting

Cell lysate of RF24 with or without annexin A2 siRNA silencing were collected and checked the expression values by Western blotting as described previously (4) using anti-

human Annexin A2 antibody (1:900) [catalog ab41803, Abcam] followed by secondary antibody conjugated with horseradish peroxidase (HRP).

Integrated network analysis (IPA®)

IPA® (QIAGEN Redwood City, www.qiagen.com/ingenuity), was used to identify pathways that were regulated by microRNAs. In short, high-throughput miRNA profiling (Nanostring) was performed using normal ovarian endothelial cells and patient tumor derived endothelial cells as described previously (5). Differentially expressed microRNAs were analyzed for gene targets using microRNA prediction software. Genes that are targets of differentially microRNAs were then analyzed for top perturbed pathways using IPA.

miRNA synthesis for tumor delivery

The anti-miR-106b-5p and anti-miR-30C-5p were synthesized on the 1 μmole scale on an Expedite 8909 DNA/RNA Synthesizer using commercially available 5′-DMT-2′-*O*-Methyl nucleoside (A^{Bz}, C^{Ac}, G^{iBu}, and U) phosphoramidite monomers, and 5′-DMT-2′-*O*-Methyl nucleoside (A^{Bz}, C^{Ac}, G^{iBu}, and U) thiophosphoramidite monomers (6-8). After completion of the synthesis, the solid support was suspended in ammonium hydroxide/methylamine (AMA) solution (prepared by mixing 1 volume of ammonium

hydroxide (28%) with 1 volume of 40% aqueous methylamine) and heated at 65 °C for 15 min to release the product from the support and to complete the removal of all protecting groups. The solid support was filtered, and the filtrate was concentrated to dryness. Purification was performed on an Amersham Biosciences P920 FPLC instrument fitted with a Mono Q 10/100 GL column (9). The anti-miRNAs were desalted with 3000 MWCO Amicon filters and lyophilized to yield the final products.

anti-miR-106b-5p 5'-A_{MS2}UC UGC ACU GUC AGC ACU $U_{MS2}U_{MS2}$A-3'

anti-miR-30c-5p 5'-G_{MS2}CU GAG AGU GUA GGA UGU UUA_{MS2} C_{MS2}A-3'

Note: MS2 stands for 2'-OMe-PS2. All the other capital letters stands for 2'-OMe-PO.

Silencing of VEGFR1 and VEGFR2 using siRNAs

RF24 cells were transfected with control or VEGFR1 or VEGFR2 siRNAs using RNAiFect transfection reagent and serum-free medium. After 6 h of transfection, MEM complete medium was added and incubated for 6 h. Cells were further incubated in the presence of VEGF (10 ng) in 2% SFM. In another set of experiments, conditioned medium from ovarian cancer SKOV3ip1 cells was added to cells after 6h of transfection to mimic the

tumor microenvironment. After 48 h of transfection, the expression of miRNAs was determined using q-RT-PCR. Experiments were done in duplicate for 3 times.

Endothelial permeability assay

To mimic the endothelium *in vivo*, we plated ECs on fibronectin-coated 3-µm pore size filters in the transwell inserts. miRNAs were silenced using miR-inhibitors by plating cells in the presence of miR-inhibitors. The tumor micro environment was created as mentioned above. After 34 h, 70-kDa FITC-dextran (5 µg/mL) was added as a permeability indicator into the upper chamber and incubated for 2 h. The permeability was quantified as fluorescence units of the FITC that passed from the upper chamber into lower chamber. Experiments were done in duplicate for 3 times.

Tube formation assay

Matrigel (12.5 mg/mL) was thawed at 4°C and 50 µL of thawed matrigel was quickly added to each well of a 96-well plate and allowed to solidify for 10 min at 37 °C. The cells (pretreated with miR-inhibitors for 36 h at conditions mimicking the tumor microenvironment) were then added to the wells and incubated for 6 h at 37°C (20,000 per well). Experiments were performed in triplicate and repeated twice. Using an Olympus IX81 inverted microscope, we obtained 5 images per well at 100x magnification. The amount of nodes (defined as when at least 3 cells formed a single point) per image was

quantified. To account for cell clumping, we removed the highest and lowest value from each group. Experiments were done in duplicate 3 times.

Synthesis and purification of a DNA library

The random single stranded DNA (ssDNA) library with a 30-nucleotide random region flanked by PCR primers was chemically synthesized using standard phosphoramidite chemistry, and the oligonucleotide strands were purified using high performance liquid chromatography under reverse-phase conditions. The library was PCR-amplified with αS-dATP to introduce the monothiophosphates, other normal dNTPs, sense primers, and 5'-biotin- labeled anti-sense primers. The ssDNA was isolated by treating the PCR products with streptavidin-coated magnetic beads and performing alkaline denaturation. The ssDNA thioaptamer (TA) library was collected in binding buffer (PBS/5 mM $MgCl_2$), heated at 95°C for 5 min and then cooled on ice to form the secondary and tertiary folds. The library was purified by 10,000 MWCO centrifugal filters (Millipore, Billerica, MA) and confirmed by gel electrophoresis (15% polyacrylamide).

Cell-based TA selection

ECs (CD31+/CD146+) had been freshly isolated from surgical samples of human normal ovaries and 10 ovarian tumors using flow cytometry (10). For the initial cycles of negative selection, we incubated ECs (1,000 cells) that had been isolated from normal ovaries with

the TA library (1µM) for 10 min at 37°C. The unbound TAs in the supernatant solution were collected and further incubated for 30 min at 37°C with ECs that had been isolated from patients with ovarian cancer. The cells were spun down to remove unbound TAs and were washed with PBS twice. The cells were suspended in 100 µL of PBS and heated at 90°C for 3 min to isolate the TAs that were bound to the cells. Subsequently, the supernatant with the bound TA fraction was collected and amplified by PCR with αS-dATP, a dNTP mix missing dATP, and biotin-labeled primers. The ssDNA was separated from the PCR products with streptavidin magnetic beads and used in the next round of selection. The selection cycles were continued for 10 rounds of cell-SELEX. The selection stringency was increased by gradually decreasing the incubation time with the ovarian cancer cells and the number of cells used in the selection.

Selection of EC-specific TAs

A panel of 7 representative TAs (out of 45) was selected after 10 rounds of cell-SELEX, and these TAs were chemically synthesized, labeled with Cy3 dye, and tested to determine whether they specifically recognized ECs of different ovarian cancer tissues. After fixing the tissue with ice-cold acetone, we incubated sections of frozen human ovarian tumors with universal blocking buffer (Pierce Biotechnology, Rockford, IL) for 30 min at ambient temperature, and then incubated them with 50 nM TAs for 20 min at

ambient temperature. The extent of TA binding to the tissue was assessed by fluorescence microscopic analysis (Nikon TE2000-E). The relative binding affinity of TAs was determined by the fluorescence intensity detected in the tumor vasculature. CD31 antibody (eBiosciences, San Diego, CA) was used as a positive control for the specific binding of ECs. Hoechst 33342 (Thermo Fisher, Waltham, MA) was used for staining nuclei. After incubating HMVECs with Cy3-labeled TAs at different concentrations (0-200nM) in 200 µL of binding buffer for 20 min at 37°C, washing, fixing, and staining with Hoechst 33324, we determined the extent of TA binding to HMVECs using fluorescence microscopy. On the basis of the binding affinity of the tested TAs to both ECs and the vasculature of human ovarian cancer tissue, we selected several TAs with high affinity and specific recognition against tumor vasculature for further analysis.

Affinity pull-down and mass spectrometry

HMVECs at 90%-95% confluence were used for the TA pull-down experiment. After being washed with cold PBS, cells were incubated with 24 nM biotinylated TAs Endo28, Endo31, or control R4 in PBS (Dulbecco's PBS with calcium chloride and magnesium chloride; Life Technologies) and gently agitated for 2 h at 4°C. After incubation, cells were cross-linked with 1% formaldehyde for 10 min at ambient temperature and quenched with glycine. Cells were harvested, washed, lysed with lysis buffer (Pierce Biotechnology), and

treated with protease inhibitors (Sigma-Aldrich). The lysates were freeze-thawed for 30 min on ice and cleared by centrifugation at 10,000 *g* for 2 min at 4°C. To pull down the labeled proteins, we incubated equal amounts of cell lysate with pre-washed streptavidin magnetic beads for 1 h at ambient temperature under continuous rotation. This approach using aptamer binding and mass spectrometry for biomarker discovery is reminiscent of the AptaBiD procedure (11). On-bead protein digestion was performed to isolate targeted proteins, and processed for mass spectrometry analysis (LTQ-Orbitrap-XL; Thermo Fisher; The University of Texas Health Science Center, Houston TX). Each sample was analyzed in triplicate. The raw data files were processed to generate a Mascot Generic Format with Mascot Distiller and searched against the SwissProt_2012_01 (Human) database using the licensed Mascot search engine v2.3.02 (Matrix Science) run on an in-house server.

Conjugation of TAs on CH and preparation of miRNA or siRNA-incorporated CH/TA-NPs

The activation and conjugation of TAs (negative control R4 and targeted Endo28) on CH were carried out as described below. TAs (negative control R4 and targeted Endo28) with free 5’-Carboxy C10 linker (Glen Research, Inc., Sterling, VA) were activated by mixing with the cross-linking agent *N*-(3-dimethylaminopropyl)-*N*′-ethylcarbodiimide

hydrochloride in the presence of *N*-hydroxysuccinimide in acetate buffer for 15 min, with spontaneous shaking to couple carboxyl groups to primary amines. CH solution was obtained by dissolving CH in 0.25% acetic acid. CH/Endo28-NPs (miRNA or siRNA) were prepared on the basis of ionic gelation of anionic TPP, miRNA or siRNA, and activated TA with cationic CH. NPs were spontaneously generated by adding TPP (0.25% w/v), miRNA or siRNA (1 µg/µL) to the CH solution (2 mg/ml) with constant stirring at ambient temperature. After incubation on ice for 30 min, activated TAs were added to the CH solution with continuous shaking and incubated for 4 h at ambient temperature. CH/TA-NPs (miRNA or siRNA) were collected by centrifugation (Thermo Biofuge, Germany) at 14,000 rpm for 40 min at 4°C. The pellet was washed 3 times to remove unbound chemicals, miRNA or siRNA, and NPs were stored at 4°C until use. Several formulations of Endo28 (1.5- 5 nmol) with CH were prepared and tested for the grafting efficiency of Endo28 on CH-NPs using native polyacrylamide gel electrophoresis with Endo28 dilutions of known concentrations as standards.

Size and zeta potential measurement of CH/Endo28-NPs (siRNA)

The size and zeta potential of the CH/Endo28-NPs (siRNA) were measured by dynamic light scattering with a particle size analyzer and Zeta Plus (Brookhaven Instruments Corporation, Holtsville, NY). NPs prepared as described previously at 2 mg/ml were

analyzed at 25°C using a back/forward scattering angle. The mean hydrodynamic diameter was calculated by cumulative analysis. The zeta potential values were determined on the basis of the electrophoretic mobility of NPs in the aqueous medium.

Gel retardation and stability assay

We used 4% agarose gel electrophoresis to analyze the NPs that were composed of TA-Endo28, siRNA, and CH. In brief, control siRNA, Endo28, CH-NPs, and CH/Endo28-NPs (containing 2.0 µg of siRNA per aptamer) were mixed with appropriate volumes of 6x gel-loading buffer and loaded on the gel. Electrophoresis was performed at a constant voltage of 100 V for 1 h in 0.5% tris-acetate EDTA buffer containing 0.5 µg/ml ethidium bromide. The siRNA bands were then visualized under an ultraviolet transilluminator (FluorChem 8900; Alpha Innotech, Madison, WI) and photographed. The stability of the CH/Endo28-NPs (siRNA) in 50% serum was characterized using 4% agarose gel electrophoresis. All test samples mentioned above were incubated with 50% serum at 37°C for 1 h followed by electrophoresis to visualize intact siRNA and TA with CH.

^{1}HNMR characterization and atomic force microscopy (AFM) of CH/Endo28-NPs

We lyophilized CH (2 mg/ml), Endo28, or CH/Endo28-NPs to remove H_2O, re-suspended them using deuterium oxide (Sigma Aldrich, St. Louis, MO), and lyophilized again. The NPs were dissolved using 5% w/v deuterium chloride solution in deuterium oxide at a

concentration of 2 mg/ml. The ^{1}H-NMR spectra were obtained using a 400-MHz Fourier transformation nuclear magnetic resonance spectrometer (Bruker Avance III; Bruker Corporation, Santa Barbara, CA).

AFM was conducted using a BioScope II™ Controller (Bruker Corporation). A drop of 10-20 µL of NP suspension at 2 mg/ml was allowed to dry on a freshly cleaved mica surface (highest grade V1 mica 12 mm discs, Ted Pella, Inc. Redding, CA) and scanned immediately after preparation. Images were acquired using the Research NanoScope software version 7.30 and analyzed using the NanoScope Analysis software, version 1.40 (Bruker Corporation). High-resolution images of CH-NPs were obtained using RTESP cantilevers (fo = 237-289 kHz, k = 20-80 N/m; Bruker Corporation). The nano-topography of the particles was determined using tapping mode operated in air to a scan rate of 0.5-0.6 Hz.

Internalization of CH/Endo28-NPs (control siRNA) into ECs

MOECs and RF24 cells were seeded onto 4-well chamber slides at 5 x 10^{4} cells per well and incubated for 48 h prior to treatment. The cells were incubated with 75 nM LysoTracker Green DND-26 (Invitrogen, Carlsbad, CA) that contained DMEM (for MOEC cells) or MEM (for RF24 cells) for 1 h to stain the lysosomes and endosomes. Next, cells were transfected with CH/Endo28-NPs (control siRNA; 160 µg NPs). 2 h after

transfection, the cells were washed with PBS, fixed using 4% paraformaldehyde, permeabilized using 0.1% Triton X-100, and stained with DAPI. Cells were then mounted with propyl gallate and visualized under an LSM 710 confocal microscope (Carl Zeiss Microscopy, LLC, Thornwood, NY) with 20x and 63x oil immersion lenses.

For the endosomal escape trafficking study, we stained MOECs with LysoTracker Green DND-26, added CH/Endo28-NPs (50 µL per well) to the cells, and incubated the cells for 0.5, 1, 2, and 4 h. Unbound NPs containing medium were removed and washed with PBS. The cells were fixed, mounted, and visualized under the confocal microscope at each pre-determined time point.

For the endocytosis pathway study, RF24 cells were treated with serum-free medium containing chlorpromazine (10 µg/ml; clathrin inhibitor), genistein (57 µg/ml; caveolin inhibitor), or both for 30 min at 37°C after the cells had been treated with LysoTracker Green DND-26. CH/Endo28-NPs were then added to cells in fresh medium to replace the

inhibitor-containing medium and incubated for 2 h. After incubation, cells were washed, fixed, mounted, and visualized under the microscope.

TA binding to ovarian tumor vasculature and therapeutic efficacy of miRNA silencing on ovarian tumors

Female athymic nude mice (NCr-nu) were purchased from the National Cancer Institute-Frederick Cancer Research and Development Center (Frederick, MD) and maintained as previously described (12). All mouse studies were approved by The University of Texas MD Anderson Cancer Center Institutional Animal Care and Use Committee, Houston. The female mice used for *in vivo* experiments were 6-8 weeks old. Detection and uptake of TAs into tumors and organs was performed as described previously (4). Relevant tissues and organs were harvested 6 h after a single intravenous injection of CH/R4-control siRNA or CH/Endo28-control siRNA (150 pmol with CH-NPs) into HeyA8-tumor-bearing mice on the eighteenth day after cell injection. Uptake efficiency was determined by the percentage of Cy3-labeled CH/Endo28-NPs located in the tissue in 5 random fields at 200X magnification for each tumor, and organ, and for macrophages. For the study of annexin A2 silencing and its influence on NP delivery to tumor vasculature, HeyA8 tumor bearing mice (on the eighteenth day after cell injection) were treated with either CH-NPs containing control siRNA or m_annexin A2 siRNA for 48 h followed by single injection of

CH/R4-NPs or CH/Endo28-NPs. Tumors were harvested 24 h after the injection of NPs and stained with anti-CD31 antibody to visualize blood vessels.

To determine the therapeutic efficacy of miRNA silencing, we used well-characterized orthotopic models of ovarian carcinoma. Ovarian tumors were established by intraperitoneally injecting HeyA8 cells (0.25×10^6 cells per 0.2 ml of Hank's balanced salt solution) into the peritoneal cavity of mice. Seven days after cell injection, mice were randomly allocated to one of the following groups: (1) CH/Endo28-control miR inhibitor, (2) CH/Endo28-control miR inhibitor + paclitaxel, (3) CH/Endo28-miR106b-5p inhibitor, (4) CH/Endo28-miR106b-5p inhibitor + paclitaxel, (5) CH/Endo28-miR30c-5p inhibitor, or (6) CH/Endo28-miR30c-5p inhibitor + paclitaxel (n = 10 mice per group). To assess tumor growth, we began treatment 1 week after cell injection. Control and targeted miR-inhibitors were incorporated into CH-NPs and intravenously injected (150 µg/kg body weight) twice weekly. Paclitaxel (35 µg/mouse) was injected once per week intraperitoneally. Mice were monitored daily for adverse effects of therapy and were sacrificed when any animals in a control or treatment group became moribund (after 3-4 weeks of therapy). At the time of sacrifice, mouse weight, tumor weight and number of tumor nodules were recorded. Individuals who performed the necropsies were blinded to the treatment group assignments. For vessel permeability, 2 mice from each group were

anaesthetized with isoflurane and injected with 100 µL of 10 mg/ml FITC-dextran (2,000,000 MW; Sigma-Aldrich) 1 hour prior to killing. Five minutes later, mice were perfused with 4% paraformaldehyde (20 ml) through the left ventricle for 5 min. Tumors were frozen in optimum cutting temperature medium (OCT; Miles, Inc., Elkhart, IN) and subsequently stained with anti-CD31 antibody for detection of blood vessels. Quantification of extravasated FITC-dextran was carried out using fluorescent microscopy under the green fluorescent filter using the following scoring system: 0 points, no staining; 1 point, <25%; 2 points, 25-50%; 3 points, 50-75%; 4 points, 75-100% FITC-dextran (using 8-10 sections per tumor at 20x magnification).

To determine whether miRNA silencing could have relevance for other tumor types, we next used an orthotopic breast cancer model (MDA-MB-231). Tumors were established by injecting 2×10^6 cells in 50 µl of Matrigel into the mammary fat pad of mice. Seven days after cell injection, mice were randomly allocated to one of the following groups: (1) CH/Endo28-control miR inhibitor, (2) CH/Endo28-control miR inhibitor + paclitaxel, (3) CH/Endo28-miR106b-5p inhibitor and (4) CH/Endo28-miR106b-5p inhibitor + paclitaxel, (n = 10 mice per group). To assess tumor growth, we began treatment 1 week after cell injection. Control and targeted miR-inhibitors were incorporated into CH-NPs and intravenously injected (150 µg/kg body weight) twice weekly. Paclitaxel (50 µg/mouse)

was injected once per week intraperitoneally. Mice were sacrificed when any animals in a control or treatment group became moribund (after 3-4 weeks of therapy). Mice were monitored daily for adverse effects of therapy and were sacrificed when any animals in a control or treatment group became moribund (after 3-4 weeks of therapy). At the time of sacrifice, mouse weight, tumor weight and number of tumor nodules were recorded. Individuals who performed the necropsies were blinded to the treatment group assignments.

Immunofluorescence staining

The binding of TAs to blood vessels and uptake of TAs by macrophages were determined as described previously (12). In brief, 5-µm frozen sections of tumors and organs were fixed in cold acetone and blocked with horse and goat sera. Tumor sections were probed with anti-CD31 (1:800) [catalog 553370, BD Pharmingen] or anti-F4/80 macrophage (1:100) [catalog MCA497G, AbD-SeroTec] primary antibodies at 4°C overnight, which was followed by incubation with Alexa 488-conjugated or Alexa 594 secondary antibodies (1:1000) [catalog 112-545-167 or 112-586-072, Jackson ImmunoResearch] for 1 h at ambient temperature and counterstained with Hoechst 33342 for 10 min. After 3 washes with PBS for 5 min, tissues were mounted with mounting medium and examined for blood vessels and macrophages. To quantify microvessel density, we recorded the number of

blood vessels that stained positive for CD31 in 5 random fields at 200X magnification for each sample. To determine the vascular stability, tumor sections were incubated with an anti-desmin antibody (pericyte marker). Pericyte coverage was determined by the percent of vessels with ≥ 50% coverage of desmin in positive cells in 5 random fields at 200X magnification of each tumor. To determine the extent of hypoxia, tumor sections were stained with a known hypoxic marker anti-CA9 (1:100) [catalog NB 100417, Novus Biologicals] antibody and hypoxic area was visualized. To quantify macrophages, we recorded the number of cells positive for macrophages in 5 random fields at the same magnification. Tumor sections were also stained for annexin A2 (1:900) [catalog ab41803, Abcam], zo1 (1:300) [catalog CST13663, Cell signaling], zo2 (1:50) [catalog CST28475, Cell signaling], claudin 5 (1:30) [catalog ab15106, Abcam] and a-SMA (1:100) [catalog ab5694, Abcam]. Staining was performed in similar way as mentioned above.

Tissue preparation and LC-MS analysis for paclitaxel

In vivo tumor tissue (about 50 mg) was weighed, homogenized in methanol/water 70%/30% solution and 15 µL of internal standard (docetaxel) solution were added. Samples were centrifuged 10 min at 15000 g, supernatant was dried in an Eppendorf vacuum concentrator and diluted consequently with 100 µL of water and 200 µL of Acetonitrile. After centrifugation at 15000 g for 10 min, clean liquid phase was transferred

to the auto sampler in high-recovery glass vial, from which 10 µL were injected for analysis. Samples were running in duplicates. Shimadzu Triple Quad 8040 (Columbia, MD, USA) with UPLC system (binary pump LC-20) was used for analysis. Compound separation were done by Restek Raptor 2.7 µm, 50 x 2.1 mm biphenyl column. The eluent was introduced to mass-spectrometer through ESI source at positive mode using Multiple Reaction Monitoring method for paclitaxel quantification. Data analysis was done with Lab Solution software (Shimadzu).

***In vivo* safety study (blood chemistry and cytokine)**

Blood samples were collected from the heart and analyzed for lactate dehydrogenase (LDH), aspartate aminotransferase (AST), alanine amino transferase (ALT), white blood cell count (WBCC), and interferon-alpha (IFN-α) and tumor necrosis factor-alpha (TNF-α) production. The cytokine levels in the supernatants were determined using ELISA kits according to the manufacturer's instructions (IFN-α: eBiosciences; TNF-α: R&D Systems, Minneapolis, MN).

***In vivo* distribution of CH/R4-NPs or CH/ Endo28-NPs**

Cy3-labeled aptamers were used to prepare CH/R4-NPs or CH/ Endo28-NPs. HeyA8 tumor-bearing nude mice were treated intravenously with 5 µg CH/R4-NPs or CH/ Endo28-NPs. Animals were sacrificed after 24 hours. Fluorescence intensity of tumor

tumor nodules and organs including kidney, spleen, liver, lung, and heart were measured using Xenogen IVIS Spectrum imaging system (535/580 nm Ex/Em filters; Caliper Life Sciences). Using Living image 2.5 software, regions of interest were drawn for each organ and the total radiant efficiency $(p\,s^{-1})/(\mu W\,cm^{-2})$ was measured.

Supplemental References

1. Sood AK, Seftor EA, Fletcher MS, Gardner LM, Heidger PM, Buller RE, Seftor RE, and Hendrix MJ. Molecular determinants of ovarian cancer plasticity. *The American journal of pathology.* 2001;158(4):1279-88.
2. Schoppmann SF, Soleiman A, Kalt R, Okubo Y, Benisch C, Nagavarapu U, Herron GS, and Geleff S. Telomerase-immortalized lymphatic and blood vessel endothelial cells are functionally stable and retain their lineage specificity. *Microcirculation.* 2004;11(3):261-9.
3. Langley RR, Ramirez KM, Tsan RZ, Van Arsdall M, Nilsson MB, and Fidler IJ. Tissue-specific microvascular endothelial cell lines from H-2K(b)-tsA58 mice for studies of angiogenesis and metastasis. *Cancer Res.* 2003;63(11):2971-6.
4. Lu C, Han HD, Mangala LS, Ali-Fehmi R, Newton CS, Ozbun L, Armaiz-Pena GN, Hu W, Stone RL, Munkarah A, et al. Regulation of tumor angiogenesis by EZH2. *Cancer Cell.* 2010;18(2):185-97.
5. Wu SY, Rupaimoole R, Shen F, Pradeep S, Pecot CV, Ivan C, Nagaraja AS, Gharpure KM, Pham E, Hatakeyama H, et al. A miR-192-EGR1-HOXB9 regulatory network controls the angiogenic switch in cancer. *Nat Commun.* 2016;7(11169.
6. Wu SY, Yang XB, Gharpure KM, Hatakeyama H, Egli M, McGuire MH, Nagaraja AS, Miyake TM, Rupaimoole R, Pecot CV, et al. 2'-OMe-phosphorodithioate-modified siRNAs show increased loading into the RISC complex and enhanced anti-tumour activity. *Nat Commun.* 2014;5(
7. Pallan PS, Yang XB, Sierant M, Abeydeera ND, Hassell T, Martinez C, Janicka M, Nawrot B, and Egli M. Crystal structure, stability and Ago2 affinity of phosphorodithioate-modified RNAs. *Rsc Adv.* 2014;4(110):64901-4.
8. Yang XB, Sierant M, Janicka M, Peczek L, Martinez C, Hassell T, Li N, Li X, Wang TZ, and Nawrot B. Gene Silencing Activity of siRNA Molecules Containing Phosphorodithioate Substitutions. *Acs Chem Biol.* 2012;7(7):1214-20.
9. Yang X, Hodge RP, Luxon BA, Shope R, and Gorenstein DG. Separation of synthetic oligonucleotide dithioates from monothiophosphate impurities by anion-exchange chromatography on a mono-q column. *Anal Biochem.* 2002;306(1):92-9.
10. Shangguan DH, Meng L, Cao ZHC, Xiao ZY, Fang XH, Li Y, Cardona D, Witek RP, Liu C, and Tan WH. Identification of liver cancer-specific aptamers using whole live cells. *Analytical Chemistry.* 2008;80(3):721-8.
11. Berezovski MV, Lechmann M, Musheev MU, Mak TW, and Krylov SN. Aptamer-facilitated biomarker discovery (AptaBiD). *Journal of the American Chemical Society.* 2008;130(28):9137-43.
12. Landen CN, Jr., Chavez-Reyes A, Bucana C, Schmandt R, Deavers MT, Lopez-Berestein G, and Sood AK. Therapeutic EphA2 gene targeting in vivo using neutral liposomal small interfering RNA delivery. *Cancer Res.* 2005;65(15):6910-8.

Figure S1. VEGF-mediated upregulation of miRNAs.

(A) Expression of miRNAs upregulated in the presence of VEGF compared with untreated control RF24 cells (n = 3). **(B)** Down-regulation of VEGFR1 and VEGFR2 using specific siRNAs (n = 3). **(C)** Silencing of either VEGFR1 or VEGFR2 receptors significantly decreased expression of both miR106b-5p and miR30c-5p in VEGF treated endothelial cells compared to untreated cells (middle section) and (n = 3). Suppression of either VEGFR1 or VEGFR2 receptors significantly decreased the expression of miR106b-5p and miR30c-5p in conditioned media treated cells compared to untreated cells (rightmost section). Cond. Media., conditioned media. Fold changes represent the mean of triplicate experiments compared to control cells. Values are means ± standard error. *$P < 0.05$ and **$P < 0.01$ (1-way ANOVA followed by a Tukey's multiple comparison post-hoc test).

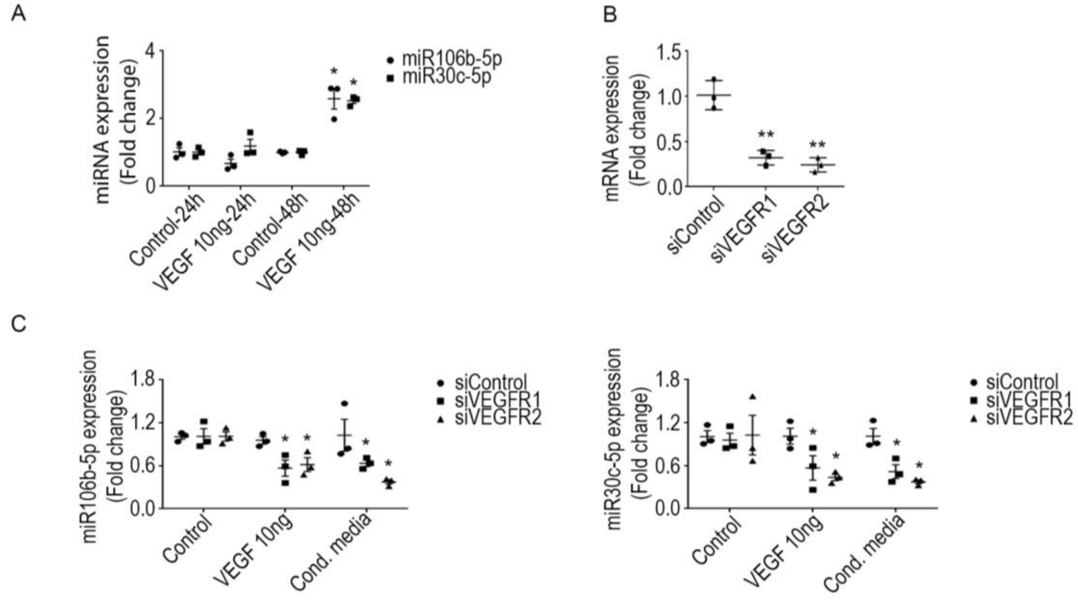

Figure S2. Integrated network analysis of dysregulated miRNAs in ovarian tumor ECs. MicroRNA array (Nanostring) was performed using normal ovarian endothelial cells (n=3) and patient tumor derived endothelial cells (n=3). Differentially expressed microRNAs were analyzed for gene targets using microRNA prediction software. Genes that are targets of differentially microRNAs were analyzed for top perturbed pathways using IPA. Bar graph depicting top ten altered cellular pathways and cellular function of miR106b-5p and miR30c-5p.

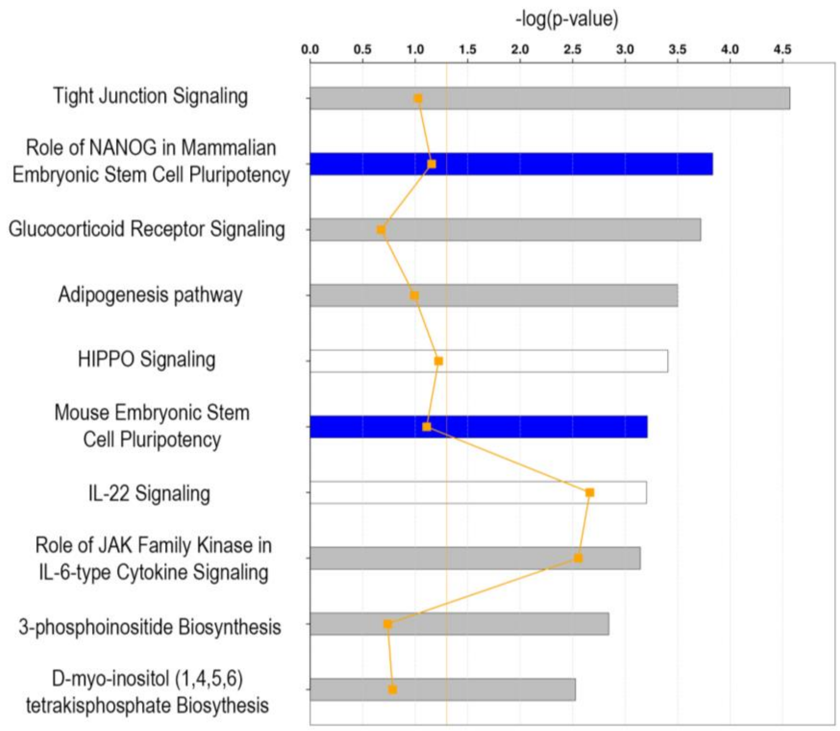

Figure S3. Silencing of miRNAs in G1S1 cells. Q-RT-PCR analysis showing specific down-regulation of miR106b-5p, miR30c-5p, and miR141-3p expression levels in G1S1 cells at 36 h treatment with targeted miR-inhibitors. Control inh., control inhibitor. Fold changes represent the mean of triplicate experiments compared to control inhibitor treated cells. Values are means ± standard error, n = 3. *$P < 0.05$ (2-tailed Student's *t* test).

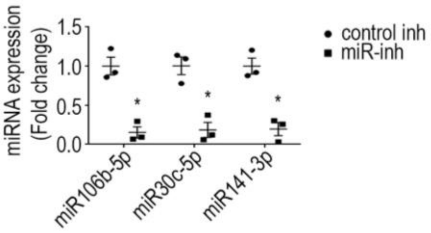

Figure S4. Sequences of Endo-TAs tested. (A) DNA sequences of Endo thioaptamers and random thioated DNA R4. Bases shown in red contain a phosphoromonothiote linkage on the 5'-side. **(B)** Endo28 and Endo31 were identified as candidate TA sequences that bind specifically to tumor ECs compared with the random TA-R4, a negative control. TAs were labeled with Cy3 in red (50 nM). Representative immunofluorescence staining of tumor sections (n = 5) showing minimal uptake of CH/Endo28-NPs by macrophages **(C)** and smooth muscle cells **(D)**. CH/Endo28., chitosan/Endo28. Nuclei are shown in blue, and CH/Endo28-NPs in red (Cy3) and macrophages or fibroblasts are shown in green. Scale bar = 100 µm in **(B)**; 50 µm in **(C)** and **(D)**.

A

Sequences of Endo-TAs tested

Endo 24	CGCTCGGATCGATAAGCTTCG-GCCCTAATCTGTCCGTTCGCCCCTCAACAG-GTCACGGATCCTCTAGAGCACTG	74
Endo 28	CGCTCGGATCGATAAGCTTCG-CTCGTCCCCCAGGCATAGATACTCCGCCCC-GTCACGGATCCTCTAGAGCACTG	74
Endo 31	CGCTCGGATCGATAAGCTTCG-TTCCCTTTACGACCACCCACGTACCACAGC-GTCACGGTCCTCTAGAGCACTG	73
Endo 36	CGCTCGGATCGATAAGCTTCG-CCGTCCATGCACTGCACCGGGCGTAGTCAC-GTCACGGATCCTCTAGAGCACTG	74
Endo 40	CGCTCGGATCGATAAGCTTCG-ATCCCACTCTCCCGTTCACTTCTCCTCAC--GTCACGGATCCTCTAGAGCACTG	73
Endo 41	CGCTCGGATCGATAAGCTTCG-CCCCACCCAACCAAACCGAGAGCCCCTGCG-GTCACGGTTCCTCTAGAGCACTG	74
Endo 42	CGCTCGGATCGATAAGCTTCG-ACCGCCCAACAACCGTCTACGCTTGAGCCC-GTCACGGATCATCTAGAGCACTG	74
R4	CCCACTTATCGTCCCTTAATGAGTTTACTCGCACACCGGACAGCCGTCGGATGGCTGGATCCGTAGCGGTCCGG	74

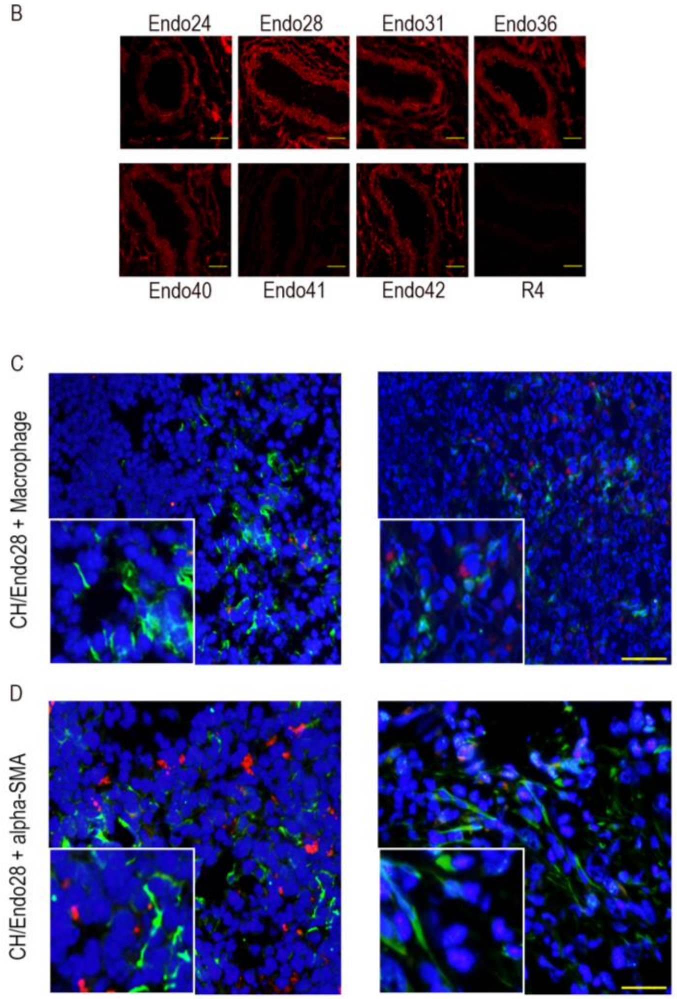

Figure S5. Annexin A2 silencing in RF24 cells. (A) Specific down-regulation of annexin A2 mRNA expression levels in RF24 using annexin A2 siRNA was confirmed by q-RT-PCR. Isolated RNA was subjected to q-real-time PCR. The fold difference in levels of annexin A2 mRNA represents the mean of triplicate experiments compared with control siRNA-treated cells. Values are means ± standard error, n = 5. **(B)** Specific knockdown of annexin A2 protein by siRNA was confirmed by Western blot analysis and densitometry. ***P* < 0.01 (2-tailed Student's *t* test).

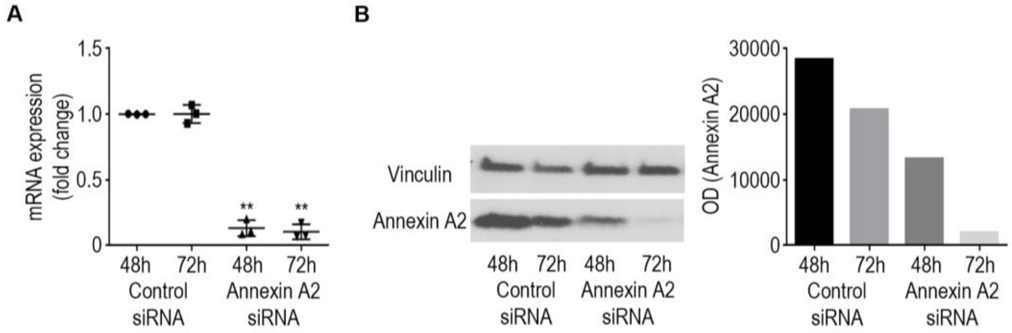

Figure S6. Physicochemical characterization of CH/Endo28-NPs. (A) Fabrication of CH/Endo28-NPs. CH is shown in green, TPP in orange, TAs in dark blue, siRNA in light blue, and Cy3 label in red. **(B)** Confirmation of grafting efficiency of Endo28 on CH-NPs by native PAGE electrophoresis (CH/Endo28-NPs) with Endo28 dilutions of known concentrations as standards. **(C)** Confirmation of conjugation of Endo28 to CH by ^{1}HNMR analysis. Spectra of CH, Endo28, and CH/Endo28 were shown separately. The spectrum of the conjugate (top one as CH/Endo28) was shown as the superposition of signals from CH and thioaptamer. The peaks at 3.5-4.0 ppm were identified as protons on the CH backbone (glucosamine and N-acetyl glucosamine monomers). The peak of around 2.0 ppm was corresponded to acetyl protons on *N*-acetyl glucosamine monomers. For Endo28, the signals above 7 ppm corresponded to the nucleotide protons. **(D and E)** Mean nanoparticle size and zeta potential values of CH/Endo28-NPs were measured by light scattering with a particle analyzer and zeta plus, showing that NPs maintained 180 to 200 nm and 22 mV. Values are means ± standard deviation, (n = 5). **(F)** Electrophoretic migration of naked siRNA, Endo28, CH/siRNA, and CH/Endo28-siRNA. CH/siRNA and CH/Endo28-siRNA (white arrow) remained at the top of the gel, in contrast with naked siRNA and Endo28 alone (red arrow), which migrated downward. **(G)** Stability of siRNA in CH/Endo28-siRNA in the presence of serum. Naked siRNA (red arrow) was degraded

after 1 h of incubation in 50% serum containing RPMI1640 medium at 37 ºC, whereas CH-NPs protected the siRNA from degradation by enzymes in serum (white arrow). **(H)** Morphology of CH/Endo28-NPs examined by atomic force microscopy (AFM) and NPs had a spherical shape. Scale bar = 1 µm. *$P < 0.05$ (1-way ANOVA followed by a Tukey's multiple comparison post-hoc test in D and E). CH., chitosan, CH/NP., chitosan/nanoparticles. All experiments were done in duplicates for 3 times (B, D to G).

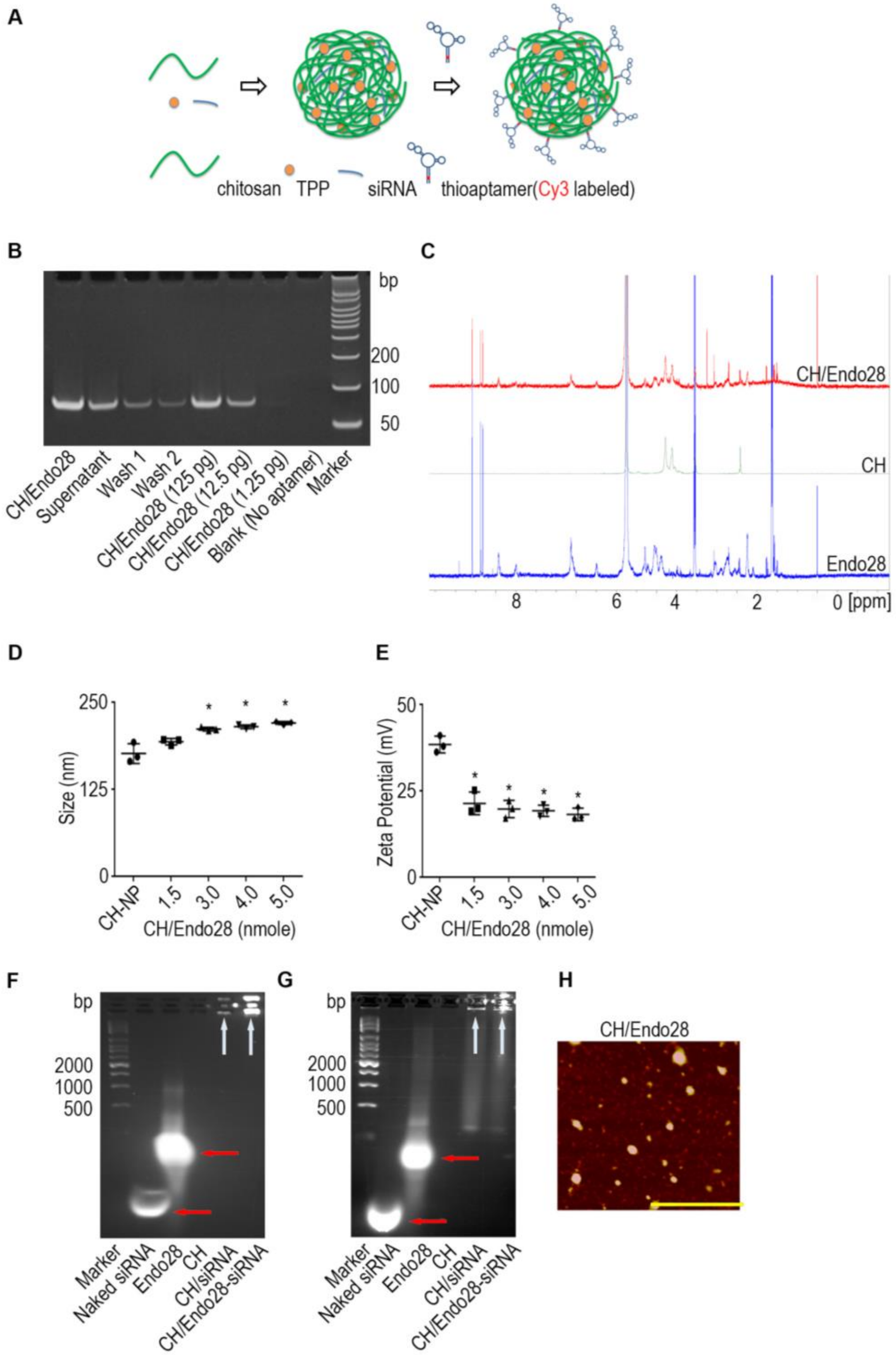
A
chitosan TPP siRNA thioaptamer(Cy3 labeled)
B
bp
200
100
50
CH/Endo28
Supernatant
Wash 1
Wash 2
CH/Endo28 (125 pg)
CH/Endo28 (12.5 pg)
CH/Endo28 (1.25 pg)
Blank (No aptamer)
Marker
C
CH/Endo28
CH
Endo28
8
6
4
2
0 [ppm]
D
Size (nm)
250
125
0
CH-NP
1.5
3.0
4.0
5.0
CH/Endo28 (nmole)
E
Zeta Potential (mV)
50
25
0
CH-NP
1.5
3.0
4.0
5.0
CH/Endo28 (nmole)
F
bp
2000
1000
500
Marker
Naked siRNA
Endo28
CH
CH/siRNA
CH/Endo28-siRNA
G
bp
2000
1000
500
Marker
Naked siRNA
Endo28
CH
CH/siRNA
CH/Endo28-siRNA
H
CH/Endo28

Figure S7. Internalization of CH/Endo28-NPs into ECs *in vitro*. (A) CH/Endo28-NPs were taken up by RF24 cells and MOECs. Cells were stained with LysoTracker green DND-26 for 1 h in serum-free medium and transfected with NPs for 2 h. **(B)** Time-dependent uptake, endosomal escape, and accumulation of CH/Endo28-NPs in MOECs. Cells were treated with LysoTracker green DND-26 for 1 h in serum-free medium and transfected with NPs for 0.5, 1, 2, or 4 h. Nuclei are shown in blue (DAPI), lysosomes in green (LysoTracker green DND-26), and NPs in red (Cy3). The yellow color in the image indicates co-localization of NPs and lysosomes. The red dots represent NPs that escaped from the lysosomes into the cytoplasm. Scale bar = 100 µm. CH/Endo28-NPs., chitosan/Endo28-nanoparticles. Both experiments were done in duplicates for 3 times (A and B).

A

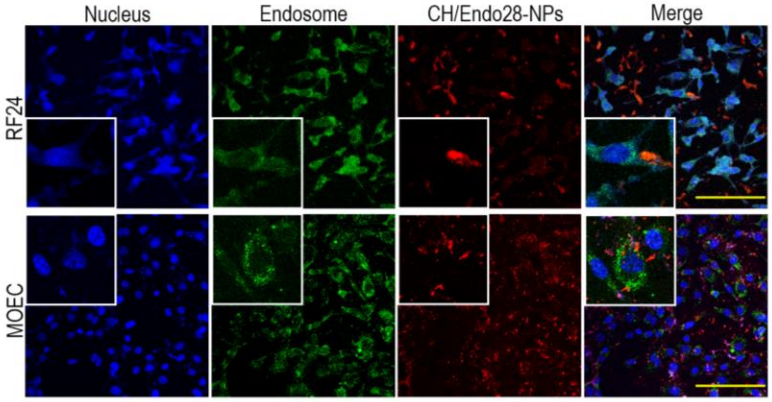
Nucleus
Endosome
CH/Endo28-NPs
Merge
RF24
MOEC

B

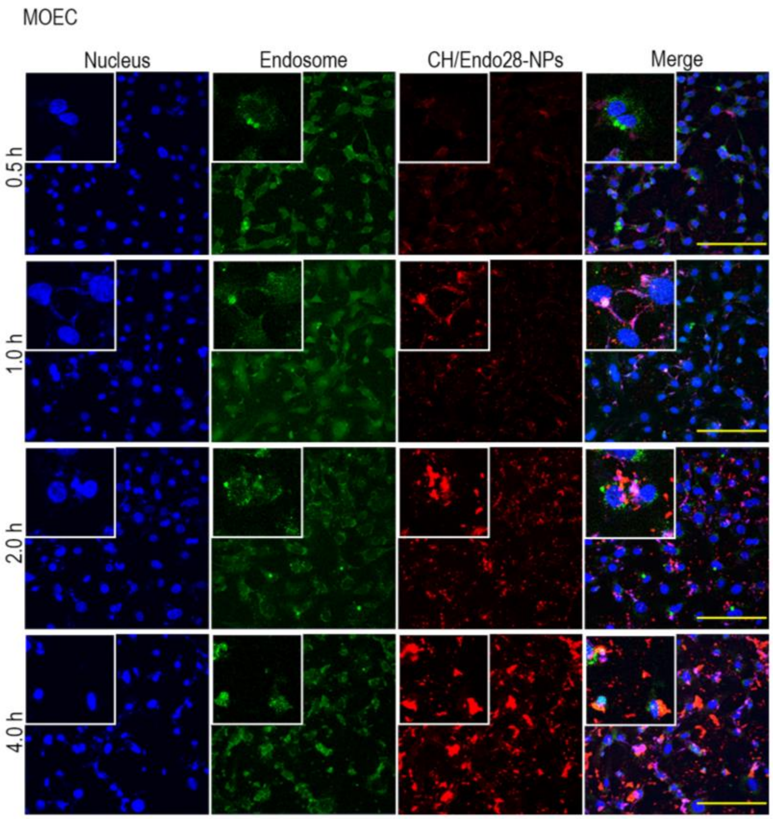
MOEC
Nucleus
Endosome
CH/Endo28-NPs
Merge
0.5 h
1.0 h
2.0 h
4.0 h

Figure S8. Mechanism of uptake of CH/Endo28-NPs into RF24 cells after treatment with clathrin (chlorpromazine) and caveolin-1 (genistein) inhibitors. RF24 cells were treated with chlorpromazine, genistein, or both for 30 min at 37°C after the cells had been treated with LysoTracker Green DND-26. CH/Endo28-NPs were then added to cells in fresh medium to replace the inhibitor-containing medium and incubated for 2 h. Nuclei are shown in blue (DAPI), lysosomes in green (LysoTracker green DND-26), and NPs in red (Cy3). Scale bar = 100 µm. CH/Endo28-NPs., chitosan/Endo28-nanoparticles. Experiment was done in duplicates for 3 times.

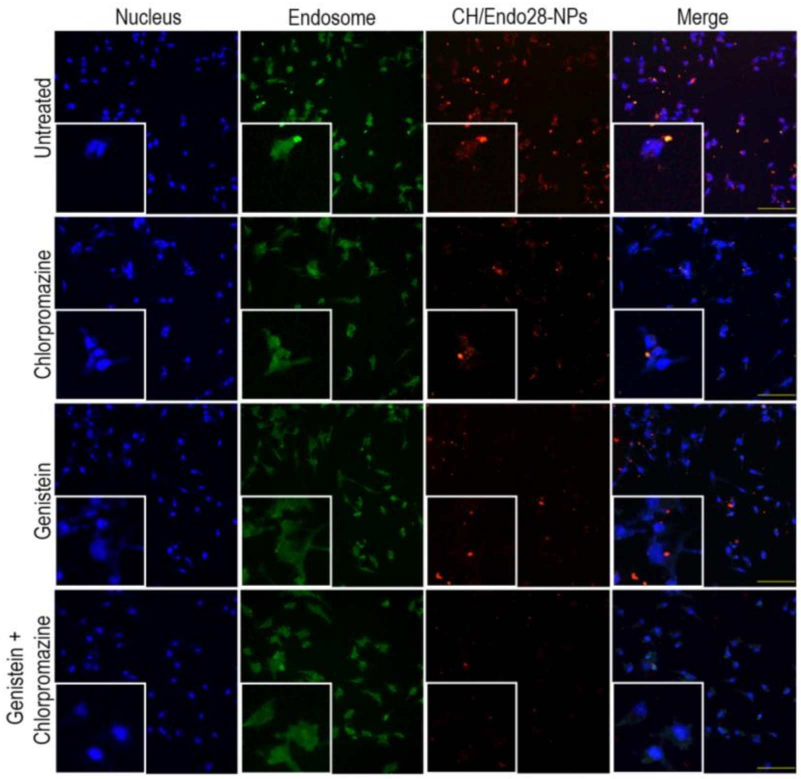

Figure S9. Delivery of CH/Endo28-NPs to tumors, organs, and macrophages. Representative fluorescent staining of tumors and organs sections which were harvested 6 h after a single intravenous injection of CH, CH/R4, or CH/Endo28-NPs into mice (150 pmol/mouse) and stained with Hoechst or anti-f4/80 antibody to detect scavenging macrophages (n = 5). Nuclei are shown in blue, and NPs in red (Cy3). The left panel represents the natural auto-fluorescence of each tissue, the middle panel represents CH/R4-NPs, and the right panel represents CH/Endo28-NPs. Macrophages are shown as surrounding nests of tumor cells (green) and have minimal siRNA uptake. Scale bar = 100 µm. CH/Endo28-NPs., chitosan/Endo28-nanoparticles.

Supplemental Figure 9

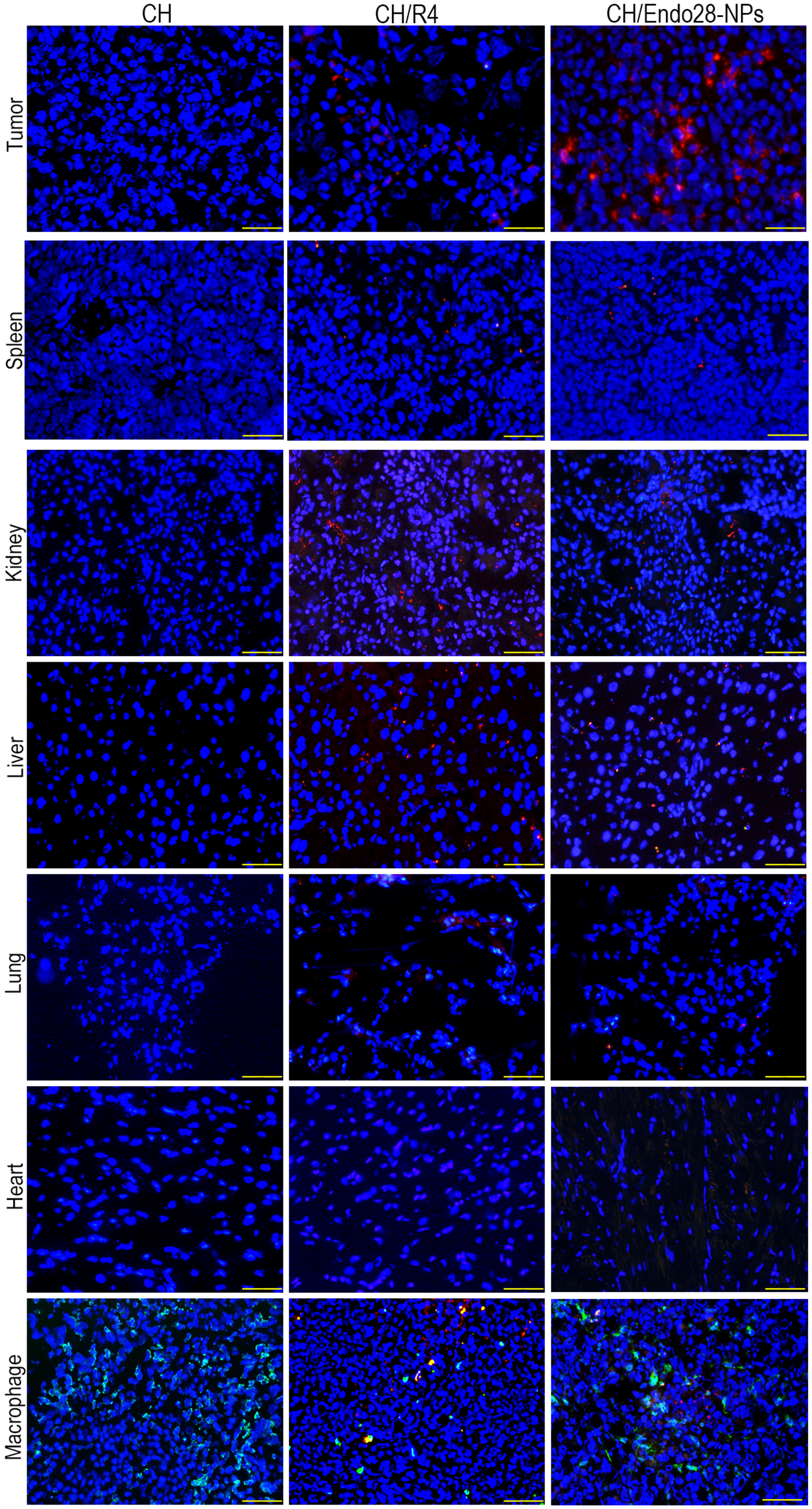

Figure S10. Expression of miRNAs in ovarian cancer cell lines. Q-RT-PCR analysis showing expression levels of miR106b-5p, miR30c-5p, and miR141-3p in G1S1 cells. Fold changes represent the mean of triplicate experiments compared to control inhibitor treated cells. Values are means ± standard error, (n = 3). **P* < 0.05 (1-way ANOVA followed by a Tukey's multiple comparison post-hoc test).

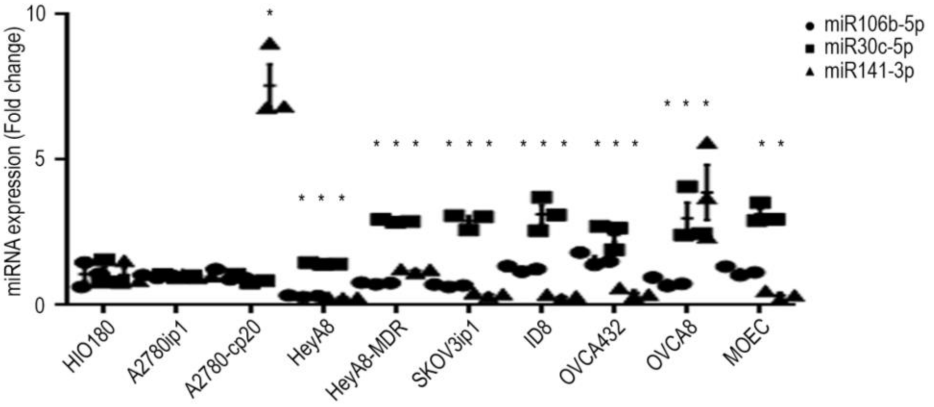

Figure S11. Therapeutic efficacy of CH/Endo28-NPs in an MDA-MB-231 breast cancer mouse model. Seven days following tumor cell injection into mammary fat pad, mice were randomly divided into 4 groups (10 mice per group) to receive 1 of the following 4 therapies: (1) CH/Endo28-control miR inhibitor, (2) CH/Endo28-control miR inhibitor + paclitaxel, (3) CH/Endo28-miR106b-5p inhibitor, (4) CH/Endo28-miR106b-5p inhibitor + paclitaxel. Mice were sacrificed when any animals in a control or treatment group became moribund (after 3-4 weeks of therapy). Representative immunofluorescence staining of tumor showing (**A**) Expression of annexin A2 in HEC1A and MDA-MB-231 tumor vasculature. (**B**) Mean tumor weight. (**C**) Mean number of tumor nodules. (**D**) Representative images of tumors from at least 5 mice per group. *$P < 0.05$ (1-way ANOVA followed by a Tukey's multiple comparison post-hoc test). Scale bar = 100 µm. miR-inh., miRNA-inhibitor.

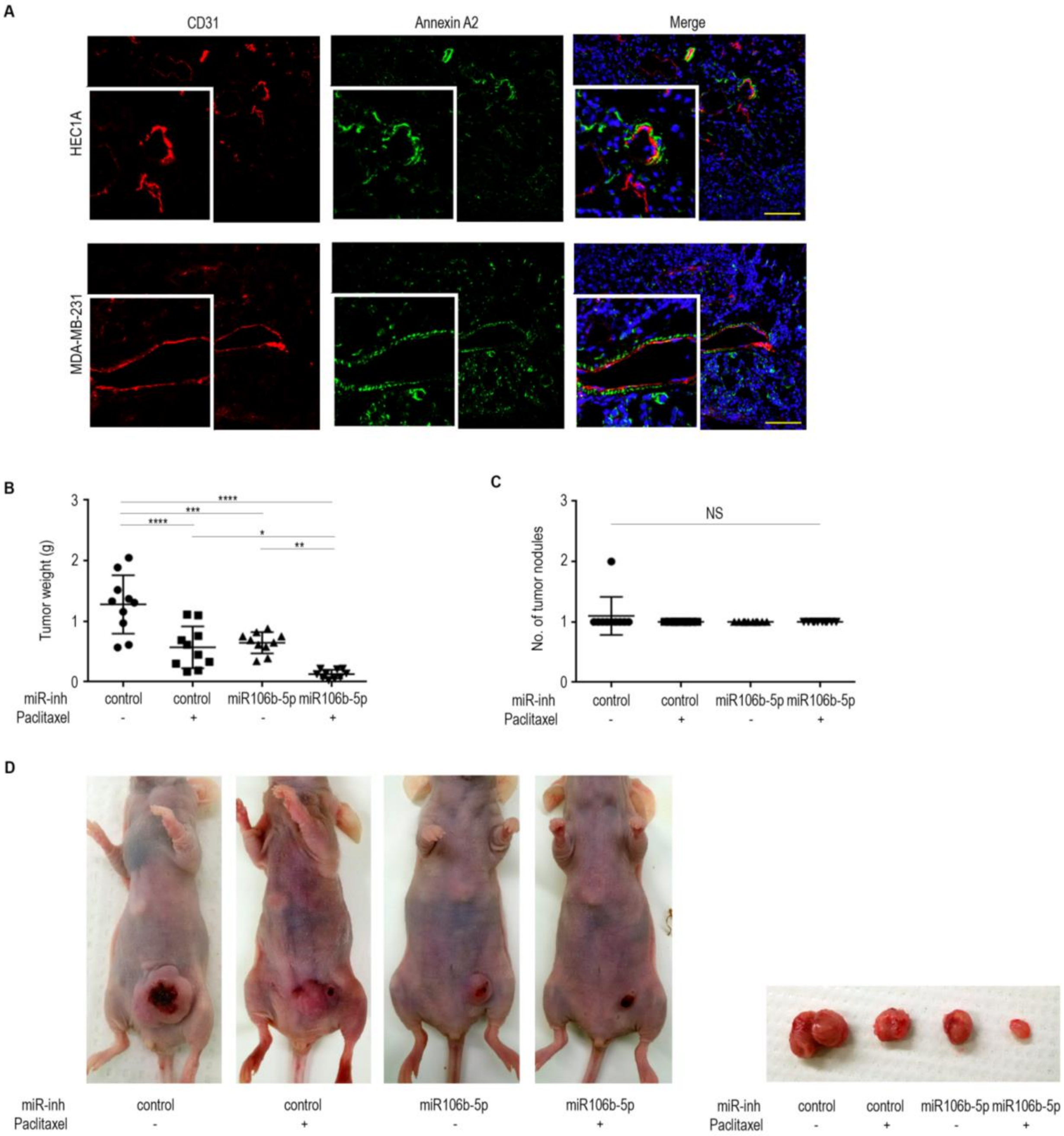
A
CD31
Annexin A2
Merge
HEC1A
MDA-MB-231
B
Tumor weight (g)
****
***
****
*
**
miR-inh
Paclitaxel
control
-
control
+
miR106b-5p
-
miR106b-5p
+
C
No. of tumor nodules
NS
miR-inh
Paclitaxel
control
-
control
+
miR106b-5p
-
miR106b-5p
+
D
miR-inh
Paclitaxel
control
-
control
+
miR106b-5p
-
miR106b-5p
+
miR-inh
Paclitaxel
control
-
control
+
miR106b-5p
-
miR106b-5p
+

Figure S12. miRNA silencing increases the expression of tight junction proteins and chemo drug delivery. Seven days following tumor cell injection, mice were randomly divided into 6 groups (9-10 mice per group) to receive 1 of the following 6 therapies: (1) CH/Endo28-control miR inhibitor, (2) CH/Endo28-control miR inhibitor + paclitaxel, (3) CH/Endo28-miR106b-5p inhibitor, (4) CH/Endo28-miR106b-5p inhibitor + paclitaxel, (5) CH/Endo28-miR30c-5p inhibitor, or (6) CH/Endo28-miR30c-5p inhibitor + paclitaxel. Mice were sacrificed when any animals in a control or treatment group became moribund (after 3-4 weeks of therapy). **(A)** Quantification of average number of blood vessels in tumor sections after indicated treatments. To quantify microvessel density, we recorded the number of blood vessels that stained positive for CD31 in 5 random fields of each section at 200X magnification for each sample. (**B**) Concentration of paclitaxel in the tumor according to liquid chromatography-mass spectrometry. Data analysis was done with Lab Solution software (Shimadzu). Values are means ± standard error (n = 10), *$P < 0.05$ (1-way ANOVA followed by a Tukey's multiple comparison post-hoc test). miR-inh., miRNA-inhibitor.

A

B

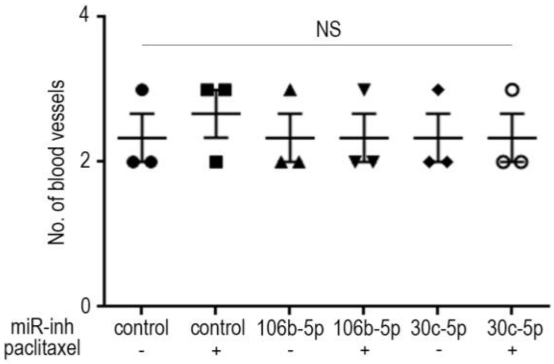

NS
No. of blood vessels
4
2
0
miR-inh
paclitaxel
control
control
106b-5p
106b-5p
30c-5p
30c-5p
-
+
-
+
-
+

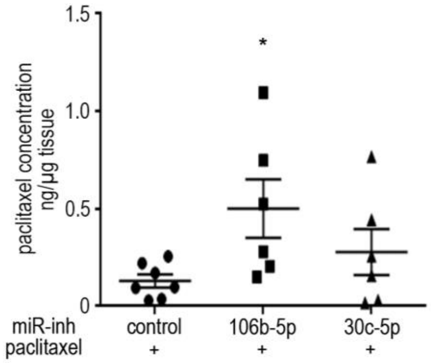

paclitaxel concentration ng/μg tissue
1.5
1.0
0.5
0
*
miR-inh
paclitaxel
control
106b-5p
30c-5p
+
+
+

Figure S13. Toxicity and immune responses of CH/Endo28-NPs in C57/BL6 mice after single intravenous injection of CH/control siRNA, CH/R4-control siRNA, or CH/Endo28-control siRNA. (A) Serum levels of AST. **(B)** Serum levels of ALT. **(C)** Serum levels of LDH. **(D)** White blood cell counts. **(E)** Serum levels of IFN-α. **(F)** Serum levels of TNF-α. Values are means ± standard error, n = 3. (1-way ANOVA followed by a Tukey's multiple comparison post-hoc test). CH., chitosan.

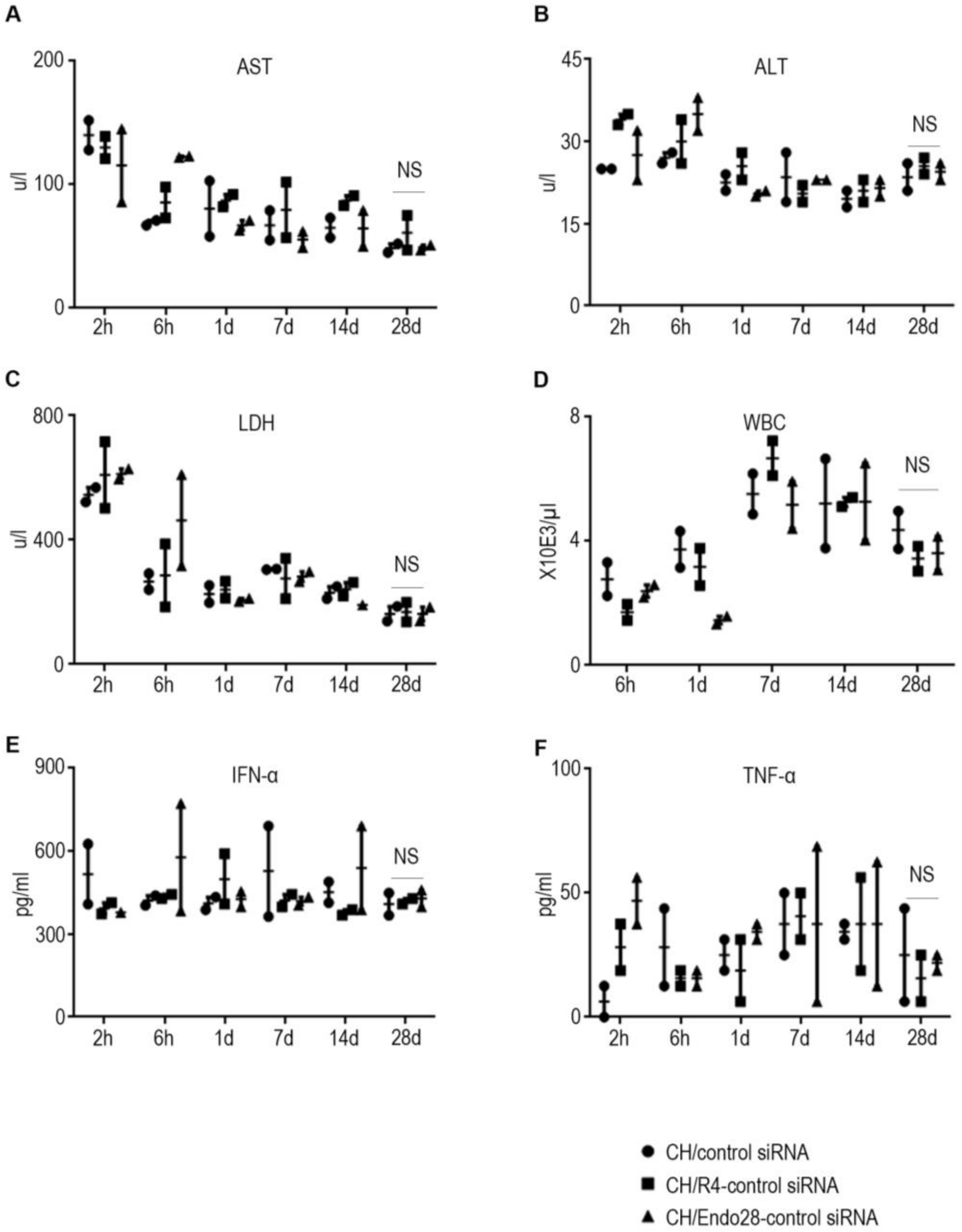

A
AST
u/l
200
100
0
2h
6h
1d
7d
14d
28d
NS
B
ALT
u/l
45
30
15
0
NS
C
LDH
u/l
800
400
0
NS
D
WBC
X10E3/μl
8
4
0
NS
E
IFN-α
pg/ml
900
600
300
0
NS
F
TNF-α
pg/ml
100
50
0
NS
CH/control siRNA
CH/R4-control siRNA
CH/Endo28-control siRNA

Supplemental Table 1. Top upregulated and downregulated miRNAs in tumor endothelial cells.

miRNA upregulated	Folds	miRNA downregulated	Folds
miR423-5p	10.1	miR100-5p	0.007
miR106b-5p	22.9	miR125b-5p	0.115
miR141-3p	14.9	miR221-3p	0.153
miR200a-3p	8.7	miR222-3p	0.006
miR30c-5p	12.1	miR24-3p	0.0856
		miR4516	0.073
		miR99a-5p	0.0721

Supplemental Table 2. miRNAs and their target tight junction proteins.

miRNA	Genes downregulated	Folds
**a.** miR30c-5p	Myosin (MYH11)	-3.8
**b.** miR30c-5p, 106b-5p & miR141-3p	MUPP1 (MPDZ)	-2
**c.** miR30c-5p	SNARE (VAMP3)	-2.6
**d.** miR141-3p	YAP1	-2.6
**e.** miR30c-5p & miR141-3p	MAGI-2	-2.2

Supplemental Table 3. Assignments of proteins pulled down by Endo28 affinity determined by mass spectrometry

Protein	Score	Mass	Matches*	Sequences*	emPAI	Description
ANXA2_HUMAN	1651	38580	67 (54)	10 (7)	11.12054	Annexin A2
H2A1B_HUMAN	633	14127	31 (25)	3 (2)	11.03565	Histone H2A type 1-B/E
H4_HUMAN	570	11360	41 (33)	2 (2)	8.319185	Histone H4
ACTG_HUMAN	535	41766	29 (15)	5 (3)	2.886248	Actin, cytoplasmic 2
ALBU_HUMAN	482	69321	54 (35)	5 (3)	1.612903	Serum albumin
TBA1A_HUMAN	314	50104	23 (11)	4 (2)	1.528014	Tubulin alpha-1A chain
LMNA_HUMAN	211	74095	5 (4)	3 (2)	1.018676	Prelamin-A/C
RAP1A_HUMAN	206	20974	10 (3)	2 (1)	1.782683	Ras-related protein Rap-1A
TBB5_HUMAN	204	49639	9 (8)	4 (3)	2.37691	Tubulin beta chain
TBB3_HUMAN	107	50400	7 (7)	2 (2)	1.528014	Tubulin beta-3 chain
SERPH_HUMAN	170	46411	5 (5)	2 (2)	1.612903	Serpin H1
MOES_HUMAN	156	67778	78 (7)	6 (2)	1.103565	Moesin
MYH9_HUMAN	146	226392	22 (7)	12 (3)	0.509338	Myosin-9
EF1A1_HUMAN	137	50109	9 (6)	4 (3)	2.37691	Elongation factor 1-alpha 1
MARCS_HUMAN	94	31536	3 (3)	1 (1)	1.188455	Myristoylated alanine-rich C-kinase substrate
RL40_HUMAN	87	14719	12 (6)	2 (1)	2.631579	Ubiquitin-60S ribosomal protein L40
RL6_HUMAN	70	32708	2 (1)	2 (1)	1.103565	60S ribosomal protein L6
ACTN1_HUMAN	69	102993	11 (4)	2 (1)	0.339559	Alpha-actinin-1
AHNK_HUMAN	63	628699	51 (5)	21 (2)	0.08489	Neuroblast differentiation-associated protein AHNAK
COF1_HUMAN	63	18491	4 (2)	2 (1)	2.037351	Cofilin-1
H14_HUMAN	54	21852	12 (2)	6 (1)	1.697793	Histone H1.4

MYL6_HUMAN	50	16919	4 (1)	2 (1)	2.207131	Myosin light polypeptide 6

*In these columns, the first number is the total count and the number in parentheses is the count for matches above the significance threshold.

Supplemental Table 4. Assignments of proteins pulled down by Endo31 affinity determined by mass spectrometry.

Protein	Score	Mass	Matches*	Sequences*	emPAI	Description
ANXA2_HUMAN	2165	38580	121 (88)	12 (9)	8.266309	Annexin A2
H4_HUMAN	1713	11360	86 (79)	2 (2)	4.37891	Histone H4
H2A1B_HUMAN	1263	14127	57 (49)	3 (2)	5.808758	Histone H2A type 1-B/E
H2A1A_HUMAN	579	14225	35 (28)	2 (2)	3.306524	Histone H2A type 1-A
MYH9_HUMAN	1174	226392	76 (38)	18 (9)	0.893655	Myosin-9
ACTB_HUMAN	939	41710	60 (35)	7 (6)	3.529937	Actin, cytoplasmic 1
ACTBL_HUMAN	578	41976	30 (16)	4 (3)	1.519214	Beta-actin-like protein 2
TBB5_HUMAN	925	49639	31 (29)	5 (4)	1.742627	Tubulin beta chain
TBB6_HUMAN	385	49825	17 (16)	3 (3)	1.251117	Tubulin beta-6 chain
LMNA_HUMAN	673	74095	21 (17)	6 (4)	1.117069	Prelamin-A/C
TBA1A_HUMAN	646	50104	29 (21)	5 (3)	1.251117	Tubulin alpha-1A chain
ACTN4_HUMAN	629	104788	21 (14)	7 (2)	0.357462	Alpha-actinin-4
RL6_HUMAN	496	32708	12 (11)	1 (1)	0.580876	60S ribosomal protein L6
MOES_HUMAN	449	67778	85 (14)	7 (3)	0.893655	Moesin
ALBU_HUMAN	407	69321	47 (27)	4 (3)	0.848972	Serum albumin
FLNB_HUMAN	395	277990	62 (20)	14 (6)	0.402145	Filamin-B
FLNA_HUMAN	232	280564	25 (11)	9 (3)	0.178731	Filamin-A
RS27A_HUMAN	388	17953	19 (16)	3 (2)	2.457551	Ubiquitin-40S ribosomal protein S27a
CLH1_HUMAN	381	191493	23 (12)	7 (2)	0.178731	Clathrin heavy chain 1
AHNK_HUMAN	320	628699	97 (19)	30 (7)	0.223414	Neuroblast differentiation-associated protein AHNAK
EF1A1_HUMAN	295	50109	16 (10)	2 (2)	1.251117	Elongation factor 1-alpha 1
RAP1A_HUMAN	280	20974	13 (5)	2 (1)	0.938338	Ras-related protein Rap-1A

H13_HUMAN	172	22336	51 (5)	5 (2)	1.921358	Histone H1.3
HNRPC_HUMAN	147	33650	5 (3)	3 (1)	0.580876	Heterogeneous nuclear ribonucleoproteins C1/C2
MYL6_HUMAN	142	16919	6 (6)	1 (1)	1.161752	Myosin light polypeptide 6
PPIA_HUMAN	141	18001	7 (7)	2 (2)	2.457551	Peptidyl-prolyl cis-trans isomerase A
RS3_HUMAN	136	26671	7 (7)	3 (3)	2.546917	40S ribosomal protein S3
RL7_HUMAN	135	29207	8 (7)	3 (2)	1.429848	60S ribosomal protein L7
RAB10_HUMAN	133	22527	5 (5)	2 (2)	1.921358	Ras-related protein Rab-10
SERPH_HUMAN	132	46411	9 (7)	3 (2)	0.848972	Serpin H1
HSP7C_HUMAN	125	70854	14 (5)	6 (2)	0.536193	Heat shock cognate 71 kDa protein
HSPB1_HUMAN	111	22768	3 (3)	1 (1)	0.848972	Heat shock protein beta-1
RS9_HUMAN	105	22578	6 (4)	1 (1)	0.848972	40S ribosomal protein S9
ML12A_HUMAN	96	19781	2 (2)	1 (1)	0.983021	Myosin regulatory light chain 12A
RLA1_HUMAN	93	11507	5 (3)	1 (1)	1.78731	60S acidic ribosomal protein P1
COF1_HUMAN	91	18491	8 (4)	5 (2)	2.368186	Cofilin-1
K2C1_HUMAN	90	65999	2 (2)	1 (1)	0.268097	Keratin, type II cytoskeletal 1
VTNC_HUMAN	80	54271	3 (2)	2 (1)	0.357462	Vitronectin
MARCS_HUMAN	77	31536	4 (3)	1 (1)	0.625559	Myristoylated alanine-rich C-kinase substrate
RS10_HUMAN	76	18886	5 (3)	2 (1)	1.027703	40S ribosomal protein S10
PRDX1_HUMAN	72	22096	4 (3)	3 (2)	1.966041	Peroxiredoxin-1
RS7_HUMAN	72	22113	4 (2)	2 (1)	0.893655	40S ribosomal protein S7
RLA2_HUMAN	68	11658	4 (3)	1 (1)	1.78731	60S acidic ribosomal protein P2
H15_HUMAN	64	22566	4 (1)	2 (1)	0.848972	Histone H1.5
ANXA1_HUMAN	61	38690	5 (2)	3 (1)	0.49151	Annexin A1
RL27A_HUMAN	54	16551	2 (2)	1 (1)	1.206434	60S ribosomal protein L27a

GBLP_HUMAN	52	35055	4 (3)	1 (1)	0.536193	Guanine nucleotide-binding protein subunit beta-2-like 1

*In these columns, the first number is the total count and the number in parentheses is the count for matches above the significance threshold.

Supplemental Table 5. Assignments of proteins pulled down by Random 4 (R4) affinity determined by mass spectrometry.

Protein	Score	Mass	Matches*	Sequences*	emPAI	Description
IQCA1_HUMAN	38	95281	120 (7)	3 (1)	0.760456	IQ and AAA domain-containing protein 1
P3C2B_HUMAN	35	184651	10 (1)	5 (1)	0.380228	Phosphatidylinositol-4-phosphate 3-kinase C2 domain-containing subunit beta
NKTR_HUMAN	31	165577	44 (4)	7 (1)	0.570342	NK-tumor recognition protein
LRCC1_HUMAN	30	119522	33 (3)	7 (1)	0.570342	Leucine-rich repeat and coiled-coil domain-containing protein 1
PRKDC_HUMAN	29	468788	95 (4)	12 (1)	0.380228	DNA-dependent protein kinase catalytic subunit
ELOV4_HUMAN	28	36805	5 (1)	1 (1)	2.281369	Elongation of very long chain fatty acids protein 4
MAP4_HUMAN	27	120930	22 (1)	3 (1)	0.570342	Microtubule-associated protein 4
MARK1_HUMAN	26	88947	43 (1)	4 (1)	0.95057	Serine/threonine-protein kinase MARK1
MLH3_HUMAN	25	163607	5 (1)	2 (1)	0.570342	DNA mismatch repair protein Mlh3
BRWD1_HUMAN	24	262772	392 (2)	14 (1)	0.380228	Bromodomain and WD repeat-containing protein 1
RN220_HUMAN	24	62725	23 (1)	2 (1)	1.330798	E3 ubiquitin-protein ligase RNF220
PCLO_HUMAN	24	552937	31 (2)	11 (1)	0.190114	Protein piccolo
VCY1_HUMAN	24	12910	11 (1)	1 (1)	6.653992	Testis-specific basic protein Y 1
PLEC_HUMAN	23	531466	414 (1)	20 (1)	0.190114	Plectin
ISL1_HUMAN	22	39010	87 (1)	1 (1)	2.091255	Insulin gene enhancer protein ISL-1
DMD_HUMAN	22	426484	40 (2)	1 (1)	0.190114	Dystrophin
TF3B_HUMAN	22	73794	52 (1)	3 (1)	1.140684	Transcription factor IIIB 90 kDa subunit
IRAK1_HUMAN	22	76489	60 (2)	1 (1)	0.95057	Interleukin-1 receptor-associated kinase 1

RL19_HUMAN	22	23451	14 (1)	2 (1)	3.612167	60S ribosomal protein L19
MYO3B_HUMAN	21	151733	10 (1)	4 (1)	0.570342	Myosin-IIIb
TT21B_HUMAN	21	150840	7 (1)	4 (1)	0.570342	Tetratricopeptide repeat protein 21B
TVA3_HUMAN	21	14699	73 (1)	1 (1)	5.893536	T-cell receptor alpha chain V region PY14
LIPS_HUMAN	20	116525	114 (1)	4 (1)	0.760456	Hormone-sensitive lipase
GA2L2_HUMAN	19	96460	19 (1)	3 (1)	0.760456	GAS2-like protein 2
CF097_HUMAN	19	82226	104 (1)	4 (1)	0.95057	Coiled-coil domain-containing protein C6orf97
DNJB1_HUMAN	19	38020	16 (2)	3 (1)	2.091255	DnaJ homolog subfamily B member 1
NBR2_HUMAN	19	12349	31 (1)	2 (1)	7.034221	Next to BRCA1 gene 2 protein
ASAP1_HUMAN	19	125420	45 (1)	5 (1)	0.570342	Arf-GAP with SH3 domain, ANK repeat and PH domain-containing protein 1
PLPL2_HUMAN	19	55281	43 (1)	1 (1)	1.520913	Patatin-like phospholipase domain-containing protein 2
UBAC1_HUMAN	19	45310	283 (1)	3 (1)	1.711027	Ubiquitin-associated domain-containing protein 1
DUS19_HUMAN	19	24179	4 (1)	2 (1)	3.422053	Dual specificity protein phosphatase 19
BAHC1_HUMAN	18	276760	9 (1)	7 (1)	0.190114	BAH and coiled-coil domain-containing protein 1
RBM6_HUMAN	18	128565	7 (1)	1 (1)	0.570342	RNA-binding protein 6
GCSP_HUMAN	18	112657	128 (1)	3 (1)	0.760456	Glycine dehydrogenase [decarboxylating], mitochondrial
NEBU_HUMAN	18	772441	73 (1)	26 (1)	0.190114	Nebulin
KIF22_HUMAN	18	73217	5 (1)	2 (1)	1.140684	Kinesin-like protein KIF22
MD13L_HUMAN	18	242447	244 (1)	7 (1)	0.380228	Mediator of RNA polymerase II transcription subunit 13-like

CN182_HUMAN	18	17437	46 (2)	3 (1)	4.942966	Uncharacterized protein C14orf182
PPR3F_HUMAN	18	82746	5 (1)	1 (1)	0.95057	Protein phosphatase 1 regulatory subunit 3F
HNRLL_HUMAN	16	60045	24 (1)	2 (1)	1.330798	Heterogeneous nuclear ribonucleoprotein L-like
NINL_HUMAN	13	156248	6 (1)	3 (1)	0.570342	Ninein-like protein

*In these columns, the first number is the total count and the number in parentheses is the count for matches above the significance threshold.
